# Severe onset of inflammatory myositis in a child: think to paraneoplastic myositis

**DOI:** 10.1186/s13052-021-01098-1

**Published:** 2021-07-01

**Authors:** Simone Benvenuto, Giulia Gortani, Rossana Bussani, Federico Poropat, Flora Maria Murru, Marco Carrozzi, Alberto Tommasini, Andrea Taddio

**Affiliations:** 1 University of Trieste, Via dell’Istria 65/1, Trieste, Italy; 2 Institute for Maternal and Child Health, IRCCS “Burlo Garofolo”, Trieste, Italy

**Keywords:** Cancer-associated myopathy, Necrotizing myopathy, Anti-TIF1- γ, Inflammatory myopathy, Teratoma, IVIG, Rituximab

## Abstract

**Background:**

Juvenile idiopathic inflammatory myopathies (JIIMs) are a group of heterogenous, acquired, autoimmune disorders that affect the muscle. While the association between IIMs and malignancy has been widely reported in adults, cancer-associated myositis (CAM) is rare in children, so that routine malignancy screening is not generally performed. This report shows a case of severe CAM in a child.

**Case presentation:**

An 11-years-old girl presented with worsening dyspnea after a 3-weeks history of progressive proximal weakness, myalgia, dysphagia, and weight loss. Her past history was remarkable for a type I Arnold-Chiari malformation associated with an anterior sacral meningocele. Physical examination showed severe hypotony and hypotrophy. Pulse oximetry and blood test showed a type II respiratory failure (SpO_2_ 88%, pCO_2_ 68 mmHg) and increased muscle enzyme levels (CPK 8479 U/L, AST 715 U/L, ALT 383 U/L, LDH 1795 U/L). The patient needed invasive mechanical ventilation. Inflammatory myositis was considered and treatment with intravenous methylprednisolone (30 mg/Kg/day for 3 days followed by 2 mg/Kg/day) and IVIG (1 g/kg/day for 2 days) was started. Muscle biopsy showed endomysial and perimysial necrosis and inflammation. The presence of serum anti-TIF1-γ antibody positivity led to a malignancy screening. Whole-body MRI showed a mature teratoma underneath sacral meningocele and both lesions were surgically removed.

Given the histological and clinical severity of the myopathy, mycophenolate (500 mg twice a day) and rituximab (360 mg/m^2^, 4 weekly infusions) were added. Due to extreme muscular wasting, severe malnutrition and intolerance to enteral feeding the patient needed a transient tracheostomy and parenteral nutrition, followed by physiotherapy, speech therapy and nocturnal non-invasive ventilation. A complete remission was achieved 3 months after.

**Conclusions:**

Among cancer-associated autoantibodies (CAAs) in adult patients, anti-TIF1-γ carries the highest risk of CAM, which recognizes with a high likelihood a paraneoplastic pathogenesis. In children, anti-TIF1-γ antibody has been associated with severe cutaneous disease, lipodystrophy, and chronic disease course, but not with CAM, which is overall rare in younger patients. Severe onset of a JIIM, especially if anti-TIF1-γ antibody positive, should prompt suspect of a CAM and lead to a screening for malignancy.

## Background

Juvenile idiopathic inflammatory myopathies (JIIMs) are a group of heterogenous, acquired, autoimmune disorders that affect muscle and, to a lesser extent, skin, with onset during childhood. Juvenile dermatomyositis (JDM) is the most recognizable and frequent (up to 95% of JIIMs), with an incidence of approximately 2.5 per million [1]; other forms of JIIMs, such as juvenile polymyositis (JP), immune-mediated necrotizing myositis (IMNM) and juvenile connective tissue disease-associated myositis (JCTM) are even rarer, and more difficult to identify compared to adult counterparts.

All of the JIIMs commonly present with an acute or subacute onset of symmetric and proximal (hip and shoulder girdles, axial muscles) weakness; typical skin manifestations (Gottron papules, heliotrope rash, V-sign and shawl-sign rashes) are key features in JDM, but usually lack in other forms of JIIM; involvement of other organ systems such as gastrointestinal tract, pulmonary system, or joints may also be present and are considered elements of disease severity [2]. Clinical diagnosis is based on EULAR/ACR classification criteria [3]. Treatment with glucocorticoids along with methotrexate is the mainstay of the therapy. More severe patients require adjunctive immunosuppressant drugs, IVIG, and/or rituximab to obtain remission.

Several myositis-associated autoantibodies (MAA) have been recognized and widely accepted in their ability to stratify patients into clinically homogenous groups so far. Their use in making an accurate diagnosis and define prognosis is emerging [4], but further characterization of their role is needed, especially in children.

Anti-transcriptional intermediary factor 1-γ (anti-TIF1-γ) antibody has been strongly correlated with cancer-associated myositis (CAM) in adults [5], but no association was found in JDM cohorts [6].

This report shows the role of anti-TIF1-γ antibody in the diagnosis of a CAM in an 11-years-old girl presenting with severe JIIM onset.

## Case presentation

An 11-years-old girl presented to the emergency department with worsening dyspnea and mild dysuria after a 3-weeks history of progressive proximal weakness to both upper and lower extremities, occasional bilateral leg myalgia, dysphagia and dysphonia. She had approximately lost the 13% of her body weight. Her past history was remarkable for incidentally diagnosed type I Arnold-Chiari malformation 5 years before, associated to a 3 cm-long cervical hydrosyringomyelia and an anterior sacral meningocele.

Physical examination was remarkable for severe diffused muscle hypotony and hypotrophy, with diminished deep tendon reflexes and abolished patellar reflexes. Pulse oximetry and capillary blood gas test showed type II respiratory failure (SpO_2_ 88%, pH 7.33, pCO_2_ 68 mmHg, HCO_3_^−^ 30 mmol/L). Blood tests were remarkable for neutrophilic leukocytosis (WBC 14.400/mm^3^, N 9.710/mm^3^) and elevated muscle enzyme levels (CPK 8479 U/L, AST 715 U/L, ALT 383 U/L, LDH 1795 U/L) with normal inflammatory markers. The neurological evaluation and a MRI scan of the brain and spine ruled out a worsening in Arnold-Chiari malformation. Capillaroscopy evaluation showed dilation and giant capillaries with avascular areas.

Findings were consistent with a severe onset of JIIM and treatment with intravenous methylprednisolone (30 mg/Kg/day for 3 days, followed by 2 mg/Kg/day) and IVIG (1 g/kg, two infusions) was started. The girl was then admitted to PICU, given her need of invasive mechanical ventilation.

**Fig. 1:**
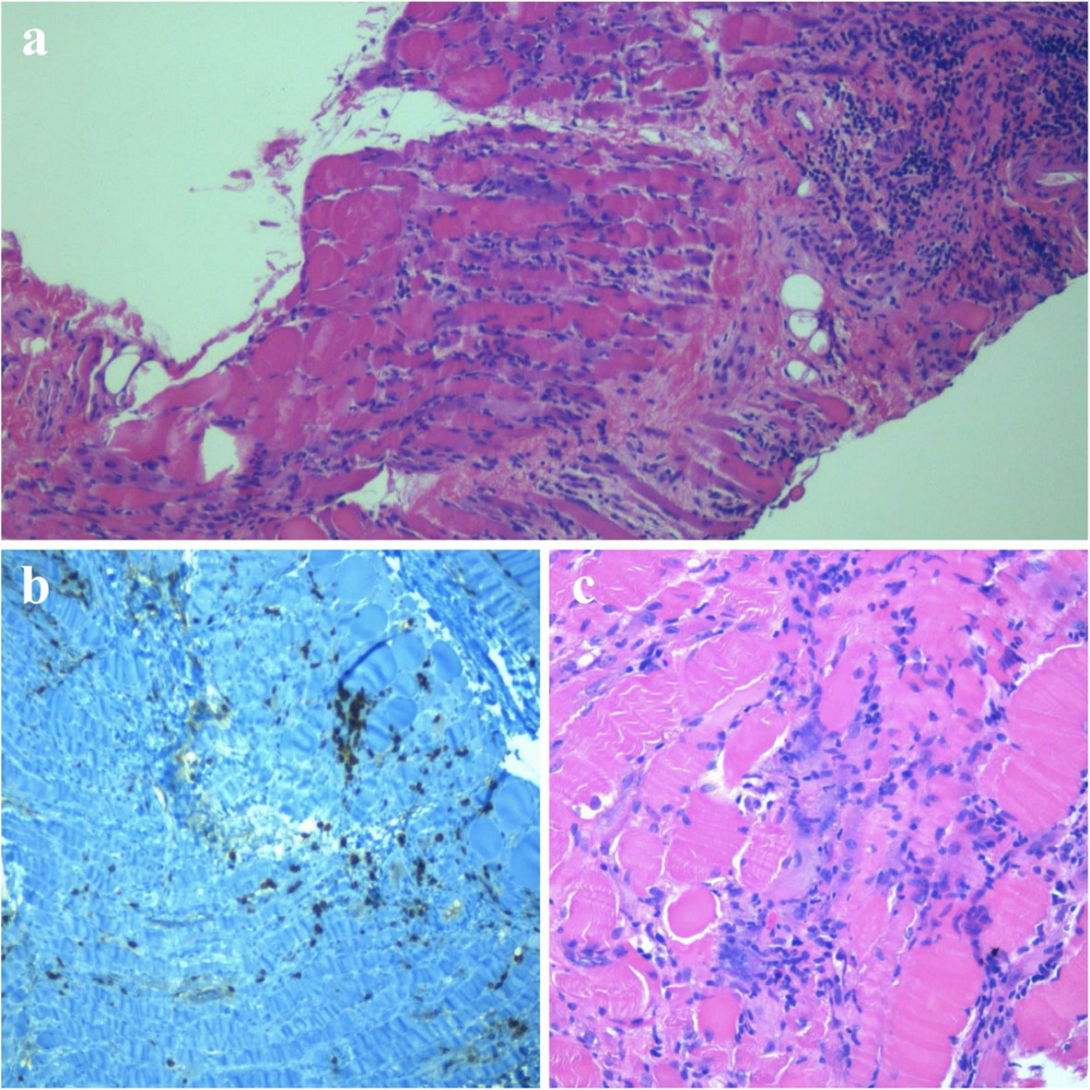
Aspects of degeneration of muscle cells with intense predominantly lymphocytic inflammation sometimes clearing parts of muscle cells

**Fig. 2:**
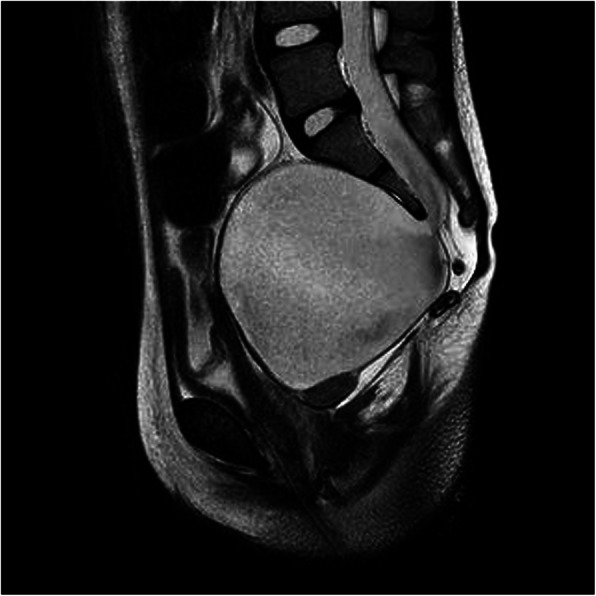
T2W MRI sagittal image showing anterior sacral meningocele (8.1 × 8.1 × 8.2 cm) and underlying teratoma (19 mm). The latter was T1W-hyperintense, and T2W- and STIR- hypointense, showing no contrast-enhancing

Metabolic myopathies were excluded through urinary organic acids and serum acylcarnitine profile evaluation. Autoantibodies screening showed ANA positivity (1:1280). Repetitive nerve stimulation test ruled out a simultaneous neuromuscular junction disorder. Muscle biopsy showed endomysial and perimysial necrosis and infiltration of mononuclear cells (CD4+ and CD8+ T-cells and NK cells) (Fig. 1) ruling out a mitochondrial myopathy. Evaluation of MAA revealed anti-TIF1-γ and anti-PM/Scl100 antibodies positivity. A Whole-body MRI showed a 19 mm-wide mass underneath the previously documented meningocele (Fig. 2). After surgical removal of both the meningocele and the mass, the latter was histologically characterized as a mature teratoma.

Due to extreme hypotonia, muscular wasting, severe malnutrition and intolerance to enteral feeding the patient needed a transient tracheostomy and parenteral nutrition, followed by physiotherapy, speech therapy and nocturnal non-invasive ventilation.

Given the clinical severity of the disease additional immunosuppressive therapy with mycophenolate mofetil (500 mg twice a day for 2 weeks, then increased to 750 mg twice a day as a result of therapeutic drug monitoring) and rituximab (360 mg/m^2^ of body surface, 4 weekly infusions) was added, together with antimicrobial prophylaxis with TMP/SMX and folic acid supplementation. Slow steroid tapering was started (5 mg decrease every week until 25 mg/day, then 2.5 mg every 10 days).

Disease activity was adequately controlled, so that muscle enzyme levels normalized from day 20 after admission (CK 139 U/L, aldolase 6.9 U/L) and a slow but progressive clinical improvement was observed. Patient was discharged from PICU at day 31.

Parenteral nutrition granted a stable weight growth until patient recovered from swallowing difficulties and incomplete glottic closure (as revealed by fiber-optic endoscopy) at day 48, when oral nutrition was restored followed by tracheostomy tube removal 5 days after.

The Childhood Myositis Assessment Scale (CMAS) [7] showed a slowly but persistent improvement in the 5 weekly measurements increasing from day 31 (CMAS: 10/52) to hospital discharge (CMAS: 35/52). Speech therapy and respiratory physiotherapy were integrated in the rehabilitation program.

Respiratory muscle function was the last to fully recover. Non-invasive ventilation was suspended at day 31, but hypoventilation persisted especially at night, as shown by overnight transcutaneous capnography performed at day 46 (average pCO2 51.9 mmHg, time over 50 mmHg: 73%; average SpO2 93%, time underneath 88%: 5%). Nocturnal non-invasive ventilation was therefore maintained for another month, when overnight capnography in spontaneous breathing confirmed recovery (average pCO2 45.1 mmHg, time over 50 mmHg: 4%).

Two months after discharge the patient was in complete clinical remission, her CMAS being 52/52. Her laboratory tests were completely normal while being treated with mycophenolate and low dosage of steroids (5 mg/day).

## Discussion and conclusions

The association between IIMs and cancer has been widely reported, with an increased risk by 2- to 7- fold in adults, so that malignancy screening is suggested for all adult patients with newly diagnosed inflammatory myositis [8]. Cancer-associated myositis (CAM) is typically defined as the development of a malignancy within 3 years of the diagnosis of myositis. Pathogenesis of CAM is still unclear, but a paraneoplastic nature has been proposed, given the cancer diagnosis and myositis onset temporal coincidence, their clinical course correlation, and common expression of myositis-specific autoantigens between cancer cells and regenerating muscle cells [9, 10]. While no significant difference was observed in the incidence of cancer among IIMs subgroups, recognized risk factors for CAM include male gender, older age at disease onset, extensive skin or muscle involvement, elevated inflammatory markers, negative ANA and/or MSAs and, interestingly, anti-SAE1, anti-NXP2, anti-HMGCR and anti-TIF1-γ antibodies positivity [10, 11], also referred to as cancer associated autoantibodies (CAAs). Adult patients with anti-TIF1-γ antibody showed the highest risk (17-fold higher compared to age- and sex-matched general population) and prevalence (40.7%) of CAM, with an estimated specificity for diagnosing CAM of 92% [12]. No correlation was found between different CAAs and certain type of cancer, prognosis (which is overall worse compared to myositis without cancer), and temporal relationship between myositis onset and cancer diagnosis [10].

In children, at least one myositis autoantibody can be identified in approximately 70% of JIIM patients [13]. Anti-TIF1-γ antibody is the most prevalent (22–36%) [4], and has been associated with more severe cutaneous disease, lipodystrophy, and chronic disease course [14], but not with CAM [6]. TIF1 family includes three 155-kDa, 140-kDa and 120 kDa proteins (TIF1-α, TIF1-β, and TIF-γ respectively), involved in several cellular pathways such as cell proliferation, apoptosis, and innate immunity [15]; in particular, high levels of TIF1-γ were found in both regenerating skeletal muscle cells [16] and tumor cells [17], supporting the hypothesis of a paraneoplastic mechanism causing CAM. Further studies are needed to explain the difference in CAM’s incidence between anti-TIF1-γ positive adult and children; the correlation between age and risk of cancer observed even among anti-TIF1-γ positive adult patients [18] could be part of the answer.

CAM is rare in children: an update by Morris [19] only found 12 pediatric cases over 45 years of literature up to 2008. Therefore, routine malignancy screening is not generally performed [20]. Nonetheless, as shown by our case, cancer can occur, defining a poorer prognosis especially if not recognized. Severe onset, with or without CAAs positivity, and anti-TIF1-γ antibody in particular, should always be considered in JIIMs and lead to perform a screening for malignancy. Anti-PM/Scl100 antibody is one of the most common MAAs in JIIMs, accounting for approximately 4% of cases; it is correlated with overlap myositis (OM) in adult patients, but data on associated clinical phenotype in children are limited [14, 21]. ANA testing does not necessarily identify a specific rheumatic disease if positive [2]; ANA positivity is found in approximately 70% of JIIM patients [22], especially if anti-TIF1-γ positive [14].

Another important issue to consider in this case is the presence of an underlying known spinal dysraphism that can be associated to cancer presence, as in our case. Benign teratomas have already been reported to be possibly associated with JIIMs, along with other paraneoplastic syndromes such as limbic encephalitis, seronegative polyarthritis, or autoimmune hemolytic anemia [23].

The treatment of CAM follows the rules of JIIM; as recently stated by SHARE recommendations [24], the mainstay of treatment is high-dose glucocorticoid (preferably methylprednisolone pulse 15–30 mg/Kg/dose for 3 days, followed by oral prednisolone 1–2 mg/Kg/day) initially in combination with methotrexate (15–20 mg/m^2^ weekly, preferably subcutaneously). Given the need of surgical intervention, in our case mycophenolate mofetil was preferred over methotrexate for its better profile in terms of infectious risk, being an effective and well tolerated option in JDM treatment as well [25]. IVIG can be added to first-line therapy of severe forms of JIIM, presenting with marked dysphagia or weakness [26]. Other treatment options, variably used for refractory disease in absence of head-to-head trials, include ciclosporin A, cyclophosphamide, azathioprine and biologics such as rituximab. In particular, in a trial with 200 adult and juvenile patients suffering from PM, DM or JDM and treated with rituximab, 83% reached a clear improvement [27], with the presence of MSAs predicting a more rapid response [28]. The severity of our patient suggested to be very aggressive in early treatment (steroids, IVIG, mycophenolate and rituximab) even if a teratoma was found and then successfully surgically removed. Steroids tapering should be considered only when clinical improvement is documented; a steroid-tapering regimen was recently proposed by PRINTO group [29], suggesting to gradually reach a prednisone dose of 1 mg/Kg/day by month 2, then the safer dose of 0.2 mg/Kg/day by month 6, and to maintain such dose up to month 12, when the dose should be halved twice more until steroid suspension at month 24. Withdrawal of disease-modifying drug should be considered once the patient is in remission and off steroids for a minimum of 1 year [24].

Overall mortality for JIIMs accounts for approximately 4%; clinical subgroup (JCTM>JPM > JDM), weight loss and dysphagia at illness onset are predictors of mortality [30]. A monocyclic course, with medication suspension within 2 years, is reported in 25% of patient, with another 25% having a polyphasic course; anti-TIF1-γ antibody positivity and severe illness onset carry a greater risk of chronic course, observed in the remaining 50% of patients [31]. Most of adult patients with CAM obtain remission after removal of malignancy, but in some cases myositis recur even without a relapse of cancer, probably because of a self-perpetuating, although cancer-triggered, immune response [12].

Severe onset of a JIIM, especially if anti-TIF1-γ antibody positive, should prompt suspect of a CAM and lead to a screening for malignancy.

## Data Availability

Data of the patient are included in the medical records of the patient.

## Abbreviations

JIIM: Juvenile Idiopathic Inflammatory Myopathy
JDM: Juvenile Dermatomyositis
JP: Juvenile Polymyositis
IMNM: Immune-Mediated Necrotizing Myositis
JCTM: Juvenile Connective Tissue disease-associated Myositis
IIM: Idiopathic Inflammatory Myopathy
MRI: Magnetic Resonance Imaging
EMG: Electromyography
MSA: Myositis Specific Autoantibodies
MAA: Myositis Associated Autoantibodies
TIF1: Transcriptional Intermediary Factor 1
CAM: Cancer-Associated Myositis
IVIG: Intravenous Immunoglobulin
SpO_2_: Peripheral Oxygen Saturation
pCO2: Partial Pressure of Carbon Dioxide
WBC: White Blood Cell
N: Neutrophils
CK: Creatine Kinase
AST: Aspartate Aminotransferase
ALT: Alanine Aminotransferase
LDH: Lactate Dehydrogenase
TMP/SMX: Trimethoprim/Sulfamethoxazole
PICU: Pediatric Intensive Care Unit
ANA: Antinuclear Antibodies
NK: Natural Killer
CMAS: Childhood Myositis Assessment Scale
DM: Dermatomyositis
SAE1: Small ubiquitin-like modifier 1 Activating Enzyme
NXP2: Nuclear Matrix Protein-2
HMGCR: Hydroxy-Methylglutaryl-Coenzyme A Reductase
CAA: Cancer Associated Autoantibody
OM: Overlap Myositis
